# Post-biopsy proteinuria as a universal prognostic marker across diverse clinical courses in IgA nephropathy

**DOI:** 10.1007/s10157-025-02808-3

**Published:** 2026-02-04

**Authors:** Akihiro Shimizu, Nobuo Tsuboi, Hiroyuki Ueda, Kentaro Koike, Masahiro Okabe, Shinya Yokote, Takaya Sasaki, Keita Hirano, Tetsuya Kawamura, Takashi Yokoo, Yusuke Suzuki, J-IGACS working group, Akihiro Shimizu, Nobuo Tsuboi, Hiroyuki Ueda, Kentaro Koike, Shinya Yokote, Takaya Sasaki, Keita Hirano, Tetsuya Kawamura, Yusuke Suzuki, Takashi Yokoo, Ryosuke Aoki, Shouichi Fujimoto, Yusuke Fukao, Akihiro Fukuda, Akinori Hashiguchi, Hiroshi Hataya, Shiko Honma, Daisuke Ichikawa, Takafumi Ito, Kensuke Joh, Ritsuko Katafuchi, Masao Kihara, Masao Kikuchi, Keiichi Matsuzaki, Kenichiro Miura, Yoichi Miyazaki, Takahito Moriyama, Kumiko Muta, Koichi Nakanishi, Shinya Nakatani, Yoshihito Nihei, Masako Nishikawa, Tomoya Nishino, Ryoko Sakaguchi, Satoru Sanada, Sayuri Shirai, Akira Shimizu, Takanori Shibata, Yuko Shima, Hitoshi Suzuki, Kazuo Takahashi, Yasuhiko Tomino, Maki Urushihara, Takashi Yasuda, Yoshinari Yasuda

**Affiliations:** 1 Division of Nephrology and Hypertension, Department of Internal Medicine, The Jikei University School of Medicine, 3-25-8 Nishi-Shimbashi, Minato-ku, Tokyo, 105-8461 Japan; 2 Department of Nephrology, Faculty of Medicine, Juntendo University, 2-1-1, Hongo, Bunkyo‑Ku, Tokyo, 113‑8421 Japan

**Keywords:** IgA nephropathy, Proteinuria, Kidney outcomes, Tonsillectomy, Glucocorticoid

## Abstract

**Background:**

Although proteinuria is a key prognostic marker in immunoglobulin A nephropathy (IgAN), the optimal post-biopsy timing for its assessment remains uncertain, particularly given variability in treatment type and timing. Using longitudinal data from the Japan IgA Nephropathy Prospective Cohort Study (J-IGACS), we sought to identify the post-biopsy time point at which proteinuria most reliably predicts kidney outcomes.

**Methods:**

Proteinuria was assessed at baseline and at 6, 12, 18, and 24 months after biopsy. The primary outcome was defined as a ≥ 50% increase in serum creatinine or initiation of kidney replacement therapy in adults (≥ 20 years) and as a ≥ 25% decline in eGFR or initiation of kidney replacement therapy in patients aged < 20 years. Model performance was compared using the corrected Akaike Information Criterion.

**Results:**

Among 588 patients (median age 38 years; mean eGFR 76.5 mL/min/1.73 m^2^; median proteinuria 0.64 g/day), 43 (7.3%) reached the primary outcome during a median 78-month follow-up. Proteinuria at all time points was independently associated with kidney outcomes, with the 18-month measurement providing the best model fit. A threshold of 0.44 g/day (or g/gCr) yielded 79% sensitivity and 81% specificity, and patients with proteinuria ≥ 0.44 g/day at 18 months had significantly worse outcomes. Cox regression confirmed a robust association for 18-month proteinuria, irrespective of treatment type or timing.

**Conclusions:**

Proteinuria measured 18 months post-biopsy showed the strongest association with long-term kidney outcomes in IgAN, supporting its use as a universal treatment target across heterogeneous post-biopsy clinical courses.

**Supplementary Information:**

The online version contains supplementary material available at 10.1007/s10157-025-02808-3.

## Introduction

Immunoglobulin A nephropathy (IgAN) is the most common form of glomerulonephritis worldwide, with a substantially higher prevalence in Asian than in non-Asian populations [1–3]. Although the initial clinical presentation is often relatively mild, the disease course can be progressive. Prior studies have revealed that approximately 30–40% of patients develop end-stage kidney disease within 20 years [4–6]. In Japanese cohorts, kidney survival rates at 10, 20, and 30 years have been reported as 84.3%, 66.6%, and 50.3%, respectively, emphasizing that IgAN is not a benign condition; long-term kidney outcomes remain poor despite available therapeutic strategies [7].

A major challenge in the management of IgAN is the considerable heterogeneity in disease progression. Among clinical parameters, proteinuria has been firmly established as a key prognostic indicator, reflecting both disease activity and therapeutic response. Persistent proteinuria is significantly associated with long-term decline in kidney function; its reduction is correlated with improved kidney outcomes. In particular, serial measurement of proteinuria provides a practical and non-invasive means of monitoring disease course and treatment effectiveness. Therefore, proteinuria serves not only as a baseline prognostic marker but also as a dynamic, time-dependent target for guiding clinical management in IgAN patients, irrespective of post-biopsy treatment status or modality. However, the optimal timing for such assessments during the post-diagnostic course remains insufficiently defined, particularly in heterogeneous real-world clinical settings where the presence and type of post-biopsy treatment considerably vary.

Building on this background, we investigated proteinuria measured between kidney biopsy and 24 months post-diagnosis in IgAN patients. Using data from a nationwide prospective cohort in Japan, we identified the specific time point at which proteinuria was most strongly associated with long-term kidney outcomes, thus providing clinically meaningful guidance for risk stratification. Our cohort is distinguished by its large sample size, longitudinal design, and inclusion of diverse initial medical interventions administered in real-world clinical practice.

## Materials and methods

### Data sources and study population

This study is a post hoc analysis of the Japan IgA Nephropathy Prospective Cohort Study (J-IGACS) [8, 9]. Patients with biopsy-confirmed primary IgAN were enrolled from multiple nephrology centers across Japan between 1 April 2005 and 31 August 2015. This analysis did not address treatment strategies; instead, it investigated the association between the duration of post-biopsy observation and long-term prognosis. Eligible patients had newly diagnosed IgAN confirmed by kidney biopsy with ≥ 10 glomeruli in the biopsy specimen. Exclusion criteria were missing proteinuria data at baseline and at 6, 12, 18, or 24 months; a follow-up duration of < 24 months; or occurrence of the primary outcome within the first 24 months.

The Ethics Review Board of the Jikei University School of Medicine approved the study protocol (36–212[12321]). Written informed consent was obtained for primary data collection, and opt-out consent was utilized for this secondary analysis. This study was conducted in accordance with the Declaration of Helsinki.

### Data collection

Clinical data were collected at baseline and every 6 months, including age, sex, mean arterial pressure (MAP), serum creatinine, estimated glomerular filtration rate (eGFR), uric acid, 24-h urinary protein excretion or urinary protein-to-creatinine ratio, and urinary red blood cell count (URBC). Proteinuria was assessed at the time of kidney biopsy (T0) and at 6 (T6), 12 (T12), 18 (T18), and 24 (T24) months thereafter, using the value closest to each time point. At T0, proteinuria was measured by 24-h urinary protein excretion (g/day); at subsequent time points, it was assessed by g/day or protein-to-creatinine ratio (g/gCr). For patients < 20 years of age, eGFR was calculated using Uemura’s equation, and for those aged ≥ 20 years at follow-up, Matsuo’s equation was applied [10, 11]. Initial treatment was defined as therapy initiated within 12 months after biopsy and was categorized as corticosteroid monotherapy (CS), corticosteroid plus tonsillectomy (CS + Tx), or conservative management (non-CS) [8]. Use of renin–angiotensin–aldosterone system inhibitors (RAASi) at the time of biopsy was also recorded as part of initial treatment. Participants were followed up until 31 May 2021.

### Definition of primary outcome

The primary outcome, consistent with the original J-IGACS definition, was a composite endpoint. For adults (≥ 20 years), this was defined as a ≥ 50% increase in serum creatinine from baseline or initiation of kidney replacement therapy. For patients < 20 years, the outcome was defined as a ≥ 25% decline in eGFR or initiation of kidney replacement therapy. Follow-up commenced at the time of biopsy and continued until event occurrence or the last available clinical assessment.

### Statistical analysis

Continuous variables are presented as mean ± standard deviation or as median with interquartile range; categorical variables are presented as frequencies and percentages. Clinical variables were compared using the Wilcoxon signed-rank test, Fisher’s exact test, or Student’s *t* test, as appropriate. All baseline variables were complete except for serum uric acid, for which mean ± standard deviation was calculated from available data. Hazard ratios (HRs) with 95% confidence intervals (CIs) were estimated using uni-variable and multivariable Cox proportional hazards models; proteinuria was treated as a continuous variable and analyzed per 1 g/day (or 1 g/gCr) increase. Model fit was evaluated using the corrected Akaike Information Criterion (AICc) and the Bayesian Information Criterion (BIC), with the model yielding the lowest AICc considered optimal. Multivariable models were adjusted for baseline covariates (age, sex, MAP, eGFR, URBC, RAASi use) and initial treatment [12]. Receiver operating characteristic (ROC) curve analysis was performed to evaluate the discriminatory ability of proteinuria for the primary outcome. Area under the curve and Youden’s index were used to determine the optimal cut-off. Kaplan–Meier curves were generated for groups above and below this cut-off; outcomes were compared using the log-rank test. Sensitivity analysis was conducted across initial treatment groups using Kaplan–Meier curves and Cox regression-based forest plots.

Sensitivity analysis was performed as follows: first, because a substantial number of patients were excluded from the primary analysis, we compared baseline clinical characteristics between the included and excluded groups. For this comparison, excluded patients were defined as those with complete baseline data for all variables except serum uric acid. Second, multiple imputation with chained equations was used to address missing proteinuria values between baseline and 24 months, as well as missing covariates used in the multivariable Cox regression analysis. 50 imputed datasets were generated under the assumption of missing at random, and each was analyzed separately using identical multivariable Cox proportional hazards models. The results were then combined using Rubin’s rules to obtain pooled hazard ratios with 95% confidence intervals and p values. Third, to explore age-related differences in outcomes, we generated Kaplan–Meier survival curves using a unified outcome definition of a ≥ 40% decline in eGFR for all patients.

Two-sided *p* values < 0.05 were considered statistically significant. HRs were reported to 1 decimal and 95% CIs to 1 decimal; when a CI boundary fell within ± 0.03 of 1.0, additional decimal places were used to avoid misinterpretation. Statistical analysis was performed using JMP Pro, version 16.2.0 (SAS Institute Inc., Cary, NC, USA). Multiple imputation and subsequent Cox proportional hazards analyses were performed using SAS software, version 9.4, with the MI, LOGISTIC, PHREG, MIXED, and MIANALYZE procedures.

## Results

### Participant characteristics

**Fig. 1:**
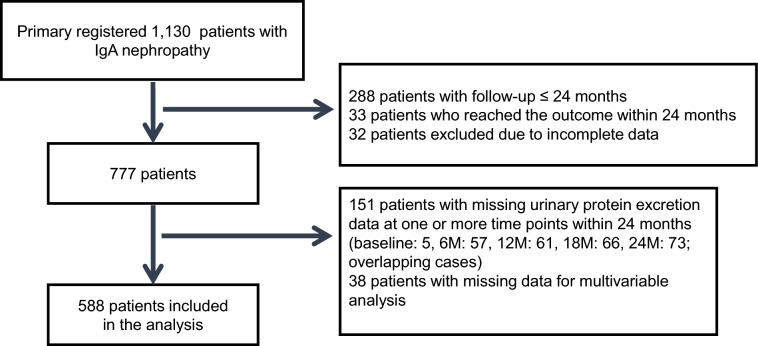
Patient enrollment flowchart. Flowchart depicting the selection of 588 patients from 1130 initially enrolled in the J-IGACS cohort, according to inclusion and exclusion criteria

Of the 1,130 patients initially enrolled, those who had a follow-up of ≤ 24 months or reached the primary outcome within 24 months, as well as those with incomplete baseline data, were excluded, leaving 777 patients. Subsequently, patients with missing proteinuria data at one or more time points between baseline and 24 months, and those with missing data required for multivariable analysis, were further excluded. The final study population comprised 588 patients (Fig. 1). Patients included in the present analysis had significantly higher T0 proteinuria levels than those excluded for incomplete data (median [IQR] 0.64 [0.30–1.27] vs. 0.49 [0.21–1.14] g/day; *p* = 0.002), whereas no significant differences were observed in any other baseline characteristics (Supplementary Table 1). Baseline characteristics and proteinuria measurements at T0, T6, T12, and T24 are summarized in Table 1. The median age was 38 years, 50.5% of patients were female, and 195 (33.2%) had hypertension. Mean eGFR was 76.5 mL/min/1.73 m^2^, and median urinary protein excretion was 0.64 g/day. The median follow-up duration was 78 months. Proteinuria exhibited a gradual decline from diagnosis (T0: 0.64 g/day) through T6 (0.32 g/day), T12 (0.21 g/day), T18 (0.19 g/day), and T24 (0.19 g/day) (Supplementary Fig. 1). Among the 588 patients analyzed, 33.5% (*n* = 197) received non-CS therapy, 26.5% (*n* = 156) were treated with CS alone, and 40.0% (*n* = 235) received CS + Tx. These distributions reflect the heterogeneity of real-world treatment practices following biopsy diagnosis. Patients in the CS + Tx group were generally younger, more often female, and less likely to have hypertension compared with those in the non-CS and CS groups. The timing of corticosteroid therapy and tonsillectomy initiation is illustrated as separate histograms in Supplementary Fig. 2, showing that most patients started treatment within 6 months after biopsy.

**Table 1:** Baseline characteristics and longitudinal proteinuria measurements during the first 2 years after diagnosis

Characteristics	Overall (*n* = 588)	Non-CS (*n* = 197)	CS (*n* = 156)	CS + Tx (*n* = 235)
*At baseline*				
Age (years)	38 (27–49)	42 (32–58)	42 (25–52)	33 (24–42)
Female, *n* (%)	297 (50.5)	93 (47.2)	75 (48.1)	129 (54.9)
MAP (mmHg)	89.5 ± 13.4	91.6 ± 13.7	90.3 ± 13.3	87.2 ± 13.0
Hypertension, *n* (%)	195 (33.2)	84 (42.6)	64 (41.0)	47 (20.0)
Diabetes mellitus, *n* (%)	10 (1.7)	2 (1.0)	5 (3.7)	3 (1.3)
eGFR (mL/min/1.73 m^2^)	76.5 ± 26.8	72.6 ± 24.4	73.3 ± 28.4	81.8 ± 26.8
URBC (0–4/5–10/11–20/21–50/> 51/HPF)	81/89/113/122/183	45/31/36/40/45	10/28/29/33/51	26/30/48/49/82
URBC ≥ 5/HPF, n (%)	507 (86.2)	152 (77.2)	146 (93.6)	209 (88.9)
URBC ≥ 20/HPF, *n* (%)	305 (51.9)	85 (43.1)	89 (57.1)	131 (55.7)
UA (mg/dL)	5.79 ± 1.52	5.90 ± 1.53	5.83 ± 1.54	5.66 ± 1.49
RAASi, *n* (%)	163 (27.8)	59 (29.9)	38 (24.4)	60 (28.1)
Follow-up				
*Proteinuria*				
T0 proteinuria (g/day)	0.64 (0.30–1.26)	0.38 (0.22–0.92)	0.89 (0.42–1.69)	0.73 (0.32–1.27)
T6 proteinuria (g/day or g/gCr)	0.32 (0.14–0.70)	0.32 (0.16–0.70)	0.24 (0.12–0.65)	0.36 (0.17–0.71)
T12 proteinuria (g/day or g/gCr)	0.21 (0.09–0.48)	0.29 (0.14–0.64)	0.17 (0.08–0.35)	0.18 (0.08–0.41)
T18 proteinuria (g/day or g/gCr)	0.19 (0.08–0.43)	0.27 (0.12–0.54)	0.18 (0.08–0.36)	0.14 (0.08–0.33)
T24 proteinuria (g/day or g/gCr)	0.19 (0.08–0.44)	0.24 (0.11–0.62)	0.21 (0.09–0.49)	0.15 (0.07–0.31)
Follow-up period (months)	78 (54–102)	84 (60–108)	78 (54–102)	78 (54–96)

Data are presented as median (interquartile range), mean ± standard deviation, or number (percentage), as appropriate

*CS* corticosteroid, *eGFR* estimated glomerular filtration rate, *HPF* high-power field, *MAP* mean arterial pressure, *RAASi* renin–angiotensin–aldosterone system inhibitors, *Tx* tonsillectomy, *UA* uric acid, *URBC* urinary red blood cell

### Association, model fit, and discriminative performance of proteinuria measurements at different time points

Among the 588 patients analyzed, 43 (7.3%) reached the primary outcome. Cox regression demonstrated that proteinuria at all time points was significantly associated with the primary outcome in both univariable and multivariable models after adjustment for confounders (Table 2). Model fit was assessed using AICc and BIC values. T18 proteinuria provided the lowest AICc and BIC, indicating the best model fit and strongest association with long-term kidney outcomes (Table 3). Furthermore, ROC curve analysis showed that T18 proteinuria had the highest area under the curve, confirming its superior discriminatory performance. The optimal cut-off for T18 proteinuria was 0.44 g/day (or g/gCr), yielding a sensitivity of 79% and a specificity of 81% (Table 4). Multiple imputation was performed as a sensitivity analysis in 777 patients, including those with missing proteinuria values at any time point up to 24 months and those with missing covariates for multivariable analysis. The effect estimates remained directionally and statistically consistent with the primary complete case analysis (Supplementary Table 2).

**Table 2:** Cox regression analysis of proteinuria parameters associated with the primary outcome

Predictors	Uni-variable model		Multivariable model	
	HR (95% CI)	*p* value	HR (95% CI)	*p* value
T0 proteinuria	1.07 (1.01–1.12)	0.003	1.05 (0.98–1.11)	0.06
T6 proteinuria	1.65 (1.29–2.00)	< 0.001	1.65 (1.26–2.07)	< 0.001
T12 proteinuria	1.12 (1.01–1.19)	0.003	1.19 (1.05–1.32)	< 0.001
T18 proteinuria	3.44 (2.67–4.37)	< 0.001	2.80 (2.11–3.70)	< 0.001
T24 proteinuria	2.41 (1.99–2.86)	< 0.001	2.15 (1.70–2.71)	< 0.001

HRs are expressed per 1 g/day or g/gCr increment in proteinuria. The multivariable model was adjusted for age, sex, MAP, eGFR, urinary red blood cell count, treatment group (non-CS, CS, and CS + Tx), and RAASi use

*CI* confidence interval, *CS* corticosteroid, *eGFR* estimated glomerular filtration rate, *HR* hazard ratio, *MAP* mean arterial pressure, *RAASi* renin–angiotensin–aldosterone system inhibitors, *Tx* tonsillectomy

**Table 3:** Model fit metrics (AICc and BIC) for proteinuria measurements at various time points in predicting the primary outcome

Model	Number of parameters	BIC	AICc	ΔAICc
T0 proteinuria	8	521.4	469.4	39.3
T6 proteinuria	8	512.6	460.7	30.6
T12 proteinuria	8	517.5	465.5	35.4
T18 proteinuria	8	482.1	430.1	0
T24 proteinuria	8	491.6	439.7	9.6

The multivariable model was adjusted for age, sex, MAP, eGFR, urinary red blood cell count, treatment group (non-CS, CS, and CS + Tx), and RAASi use

*AICc* corrected Akaike information criterion, *BIC* Bayesian information criterion, *CS* corticosteroid, *eGFR* estimated glomerular filtration rate, *MAP* mean arterial pressure, *RAASi* renin–angiotensin–aldosterone system inhibitors, *Tx* tonsillectomy

**Table 4:** Discriminative performance and optimal cut-off values of proteinuria measurements at various time points

Model	AUC	Cut-off value (g/day or g/gCr)	Sensitivity (%)	Specificity (%)
T0 proteinuria	0.72	0.70	86	56
T6 proteinuria	0.72	0.62	70	73
T12 proteinuria	0.82	0.29	88	66
T18 proteinuria	0.85	0.44	79	81
T24 proteinuria	0.84	0.61	79	86

*AUC* area under the curve

### Kaplan–Meier analysis stratified by the threshold of T18 proteinuria

**Fig. 2:**
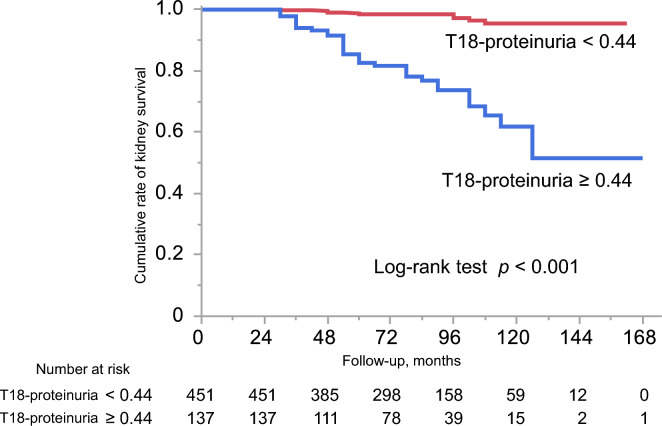
Kaplan–Meier curves stratified by T18 proteinuria. Kaplan–Meier curves showing the cumulative incidence of the primary outcome in patients with T18 proteinuria ≥ 0.44 g/day (or g/gCr) versus < 0.44 g/day (or g/gCr). The high-proteinuria group demonstrated a significantly greater incidence of the primary outcome (log-rank *p* < 0.001)

Kaplan–Meier survival curves stratified by T18 proteinuria are presented in Fig. 2. Patients with T18 proteinuria ≥ 0.44 g/day (or g/gCr) had a significantly higher incidence of the primary outcome relative to those with < 0.44 g/day (log-rank *p* < 0.001). To assess consistency across treatment strategies, patients were further stratified into non-CS, CS, and CS + Tx groups. Numbers of patients reaching the primary outcome were 23 (11.7%) in the non-CS group, 14 (9.0%) in the CS group, and 6 (2.6%) in the CS + Tx group. Kaplan–Meier curves demonstrated that higher T18 proteinuria (≥ 0.44 g/day) was consistently associated with significantly worse outcomes across all treatment groups (Supplementary Fig. 3). As a sensitivity analysis, Kaplan–Meier survival curves were generated using a unified outcome definition of a ≥ 40% decline in eGFR for both adult and pediatric patients. The results were nearly identical to those of the primary analysis (Supplementary Fig. 4).

### T18 proteinuria and prognosis across initial treatment groups

**Fig. 3:**
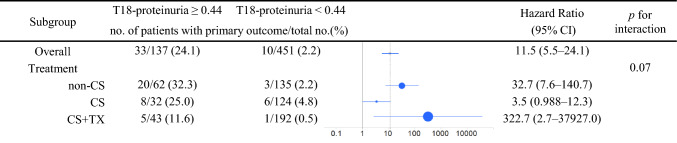
Multivariable Cox regression of T18 proteinuria for the primary outcome. Forest plot illustrating the hazard ratio (HR) with 95% confidence intervals (CIs) for T18 proteinuria (≥ 0.44 vs. < 0.44 g/day or g/gCr) in the overall cohort and stratified by initial treatment group (non-corticosteroid [non-CS], corticosteroid [CS], and corticosteroid plus tonsillectomy [CS + Tx]). Models were adjusted for age, sex, mean arterial pressure (MAP), estimated glomerular filtration rate (eGFR), urinary red blood cell count (URBC), and renin–angiotensin–aldosterone system inhibitors (RAASi) use. No significant interaction was detected between T18 proteinuria and treatment group (*p* for interaction = 0.07)

In the overall cohort, T18 proteinuria ≥ 0.44 g/day (or g/gCr) was significantly associated with an increased risk of adverse kidney outcomes (HR 11.5, 95% CI 5.5–24.1). Subgroup analysis stratified by initial treatment (non-CS, CS, and CS + Tx) demonstrated consistent trends although the strength of association differed across groups. No statistically significant interaction was observed between treatment group and T18 proteinuria (*p* for interaction = 0.07), indicating no evidence of effect modification by initial treatment (Fig. 3).

## Discussion

In this post hoc analysis of a prospective cohort of IgAN patients, we identified the optimal timing and cut-off value of proteinuria significantly associated with kidney prognosis. Proteinuria measured at 6-month intervals from baseline to 24 months was evaluated as a candidate prognostic marker. Among these, proteinuria at 18 months demonstrated the best model fit as reflected by the lowest AICc value. The optimal cut-off, derived from ROC analysis, was 0.44 g/day (or g/gCr). T18 proteinuria ≥ 0.44 g/day (or g/gCr) was significantly associated with adverse kidney outcomes; this association was consistent across treatment groups, with no evidence of effect modification. These findings emphasize the prognostic value of proteinuria measured after diagnostic intervention and support a management target of < 0.44 g/day (or g/gCr) at 18 months.

Our findings indicate that proteinuria measured 18 months after diagnosis is significantly associated with kidney prognosis, irrespective of the initial treatment strategy. Although baseline proteinuria is a well-established prognostic factor, reliance on diagnostic values alone may underestimate the benefits of early intervention [13–16]. Accordingly, the prognostic significance of post-treatment proteinuria has gained increasing support from accumulating evidence. Prior studies have demonstrated that proteinuria at 12 months predicts kidney function decline, and remission within 2 years has been associated with favorable outcomes [17–19]. Although more dynamic approaches, such as time-varying proteinuria and time-averaged proteinuria, reflect cumulative burden and trajectory, they are less feasible for routine practice due to their complexity [20–22]. Recent studies have emphasized the prognostic value of proteinuria reduction at 6 or 9 months as an early surrogate for long-term outcomes [23, 24]. However, such reduction-based endpoints may be less applicable to our cohort, in which median baseline proteinuria was relatively low (0.65 g/day, compared with 1.0–2.5 g/day in previous reports). Notably, our analysis was structured around fixed post-biopsy time intervals, independent of treatment modality. In our cohort, initial therapies, such as corticosteroids and tonsillectomy, were most frequently initiated within 6 months of diagnosis, with a second peak around 12 months, and proteinuria levels reached their lowest values at 18 months in the CS and CS + Tx groups. These treatment-related dynamics likely contributed substantially to the overall finding that T18 proteinuria showed the strongest association with long-term kidney outcomes in the entire cohort. Although no significant interaction was observed between treatment type and timing of response, these findings emphasize the potential value of a time-based approach to outcome assessment, particularly in real-world settings where therapeutic strategies are heterogeneous.

Our data indicate that T18 proteinuria ≥ 0.44 g/day (or g/gCr) is significantly associated with adverse kidney outcomes. Although numerous earlier studies used a 1 g/day threshold, more recent studies have suggested that lower levels (e.g., > 0.3 g/day) are clinically relevant [13–16]. Hirano et al. reported that proteinuria < 0.4 g/day at 12 months predicted favorable outcomes in patients treated with corticosteroids, including a 6-month pulse regimen [18]. Pitcher et al. demonstrated that the 10-year risk of kidney failure increased even among patients with time-averaged proteinuria of 0.44–0.88 g/gCr [25]. Tang et al. found that time-varying proteinuria ≥ 0.5 g/day was associated with kidney failure [20]. Collectively, these findings place prognostically meaningful thresholds within the 0.3–0.5 g/day (or g/gCr) range, supporting the validity of our 0.44 g/day cut-off. Taken together, the evidence emphasizes the utility of 0.44 g/day as a robust threshold for defining post-biopsy disease stabilization and predicting long-term kidney prognosis, regardless of treatment strategy.

Our findings were derived from an integrated analysis across all initial treatment groups. Although the same proteinuria cut-off (≥ 0.44 g/day or g/gCr) exhibited consistent prognostic trends, no statistically significant interaction was detected. However, the optimal timing and the threshold of proteinuria measurement may differ according to treatment modality. Due to the limited sample sizes in each subgroup and the low incidence of kidney outcomes, stratified analysis with multivariable adjustment was not feasible. Larger studies are needed to determine whether treatment-specific cut-offs should be considered. Furthermore, the 18-month time point in our study does not necessarily represent a fixed interval from treatment initiation, given that therapy timing and intensity varied across patients. Despite this variability, the consistent association between T18 proteinuria and kidney outcomes emphasizes its potential as a robust, time-sensitive prognostic marker that reflects the cumulative impact of disease activity and treatment response over time.

Proteinuria is both a marker of ongoing kidney injury and a pathogenic driver of disease progression. In IgAN, it primarily results from glomerular inflammation and injury triggered by mesangial IgA deposition. This injury disrupts the filtration barrier, leading to urinary protein loss. As glomerulosclerosis advances, nephron loss induces compensatory hemodynamic changes, including glomerular capillary hypertension, which further impair the filtration barrier and increase protein leakage. Proteinuria in turn promotes tubular injury and interstitial inflammation, accelerating fibrosis and chronic kidney disease [26]. These mechanisms emphasize its dual role as both biomarker and mediator of kidney injury. In our study, proteinuria measured at all predefined time points was significantly associated with kidney prognosis. This pathophysiological basis supports the use of proteinuria reduction as a surrogate marker of treatment response in IgAN. Therefore, defining the optimal timing and threshold for its prognostic application is clinically important because clear proteinuria-based criteria can improve risk stratification and inform therapeutic decision-making.

Our study had several limitations. First, patients with missing proteinuria data up to 24 months, follow-up duration < 24 months, early primary outcomes, or biopsy specimens containing < 10 glomeruli were excluded. T0 proteinuria levels were significantly higher in the inclusion group than in the exclusion group, whereas age, kidney function, and other baseline characteristics were comparable between the two groups. The reason for and clinical significance of this difference in baseline proteinuria levels remain unclear; however, it is possible that excluded patients had milder disease that did not require treatment, and these patients may have discontinued follow-up at an early stage. These exclusions may therefore have yielded a more homogeneous cohort, potentially limiting generalizability. Second, proteinuria was assessed using both g/day and g/gCr units, which may have introduced variability and reduced comparability across patients. The absence of standardized measurement protocols may also limit the clinical applicability of our findings. Third, the number of outcome events was relatively small, restricting statistical power to assess long-term risks, particularly among low-risk patients. Finally, although the nationwide design enhances applicability to the Japanese IgAN population, generalizability to other populations remains uncertain.

Our findings indicate that proteinuria measured at 18 months after diagnosis was more strongly associated with kidney prognosis in IgAN than were measurements obtained at other time points within the first 24 months. To our knowledge, this is the first prospective cohort analysis to statistically validate 18-month proteinuria as the optimal prognostic marker in IgAN. A T18 proteinuria threshold of < 0.44 g/day or g/gCr represents a practical and clinically relevant target associated with favorable long-term kidney outcomes. Notably, this threshold was predictive irrespective of subsequent treatment presence or modality, supporting its applicability across diverse clinical contexts. Accordingly, this cut-off may serve as a universal treatment goal during post-biopsy follow-up in IgAN patients.

## Supplementary Information

Below is the link to the electronic supplementary material.

Supplementary file1 (DOCX 340 kb)

## Data Availability

The data underlying this article are available from the corresponding author upon reasonable request.
