# Supplementary material for: Post-biopsy proteinuria as a universal prognostic marker across diverse clinical courses in IgA nephropathy
Source: Clin Exp Nephrol. 2026 Feb 4;30(3):498–506. doi: 10.1007/s10157-025-02808-3 (PMC12950100; doi:10.1007/s10157-025-02808-3)
Supplement: Supplementary file 1 — Supplementary file1 (DOCX 340 kb) [file 10157_2025_2808_MOESM1_ESM.docx]

**Supplementary Material**

**Supplementary Table 1** Baseline characteristics of included versus excluded patients in the present study

**Supplementary Table 2** Multivariable Cox regression analysis of proteinuria parameters associated with the primary outcome after multiple imputation (n = 777)

**Supplementary Fig. 1** Longitudinal trajectories of proteinuria

**Supplementary Fig. 2** Distribution of initiation timing for corticosteroid therapy and tonsillectomy after kidney biopsy

**Supplementary Fig. 3** Kaplan–Meier survival curves stratified by initial treatment groups and T18-proteinuria

**Supplementary Fig 4** Kaplan–Meier curves stratified by T18-proteinuria for a ≥ 40% decline in eGFR

**Supplementary Table 1** Baseline characteristics of included versus excluded patients in the present study.

| Characteristics | Included patients (n = 588) | Excluded patients  (n = 438)* | *p* value |
| --- | --- | --- | --- |
| Age (years) | 38 (27–49) | 36 (25–54) | 0.96 |
| Female, n (%) | 297 (50.5) | 220 (50.2) | 0.95 |
| MAP (mmHg) | 89.5 ± 13.4 | 91.1 ± 14.1 | 0.07 |
| Hypertension, n (%) | 195 (33.2) | 165 (37.8) | 0.13 |
| Diabetes mellitus, n (%) | 10 (1.7) | 11 (2.5) | 0.38 |
| eGFR (mL/min/1.73 m^2^) | 76.5 ± 26.8 | 75.7 ± 31.9 | 0.7 |
| URBC (0–4 / 5–10 / 11–20/ 21–50 / > 51/HPF) | 81/89/113/122/183 | 65/56/90/98/129 | 0.75 |
| T0-proteinuria (g/day) | 0.64 (0.30–1.27) | 0.49 (0.21–1.14) | 0.002 |
| UA (mg/dL) | 5.79 ± 1.52 | 5.95 ± 1.82 | 0.14 |
| RAASi, n (%) | 163 (27.7) | 122 (28.1) | 0.94 |

Data are presented as median (interquartile range), mean ± standard deviation, or number (percentage), as appropriate.

eGFR, estimated glomerular filtration rate; HPF, high-power field; MAP, mean arterial pressure; RAASi, renin–angiotensin–aldosterone system inhibitors; UA, uric acid; URBC, urinary red blood cell.

* Among the excluded patients, those with missing baseline clinical data (excluding UA) were further removed, leaving 438 patients from the initial 542.

**Supplementary Table 2** Multivariable Cox regression analysis of proteinuria parameters associated with the primary outcome after multiple imputation (n = 777).

| Predictors | HR (95% CI) | *p* value |
| --- | --- | --- |
| T0-proteinuria | 1.06 (1.00–1.12) | 0.04 |
| T6-proteinuria | 1.72 (1.39–2.14) | < 0.001 |
| T12-proteinuria | 1.17 (1.17–1.27) | < 0.001 |
| T18-proteinuria | 2.89 (2.23–3.74) | < 0.001 |
| T24-proteinuria | 2.17 (1.77–2.67) | < 0.001 |

HRs are expressed per 1 g/day or g/gCr increment in proteinuria. The multivariable model was adjusted for age, sex, MAP, eGFR, urinary red blood cell count, treatment group (non-CS, CS, and CS+Tx), and RAASi use.

CI, confidence interval; CS, corticosteroid; eGFR, estimated glomerular filtration rate; HR, hazard ratio; MAP, mean arterial pressure; RAASi, renin–angiotensin–aldosterone system inhibitors; Tx, tonsillectomy.

**Supplementary Fig. 1** Longitudinal trajectories of proteinuria.

Proteinuria levels at baseline and at 6, 12, 18, and 24 months: (a) overall cohort, and (b) stratified by initial treatment group. CS, corticosteroid; Tx, tonsillectomy; UPE, urinary protein excretion.


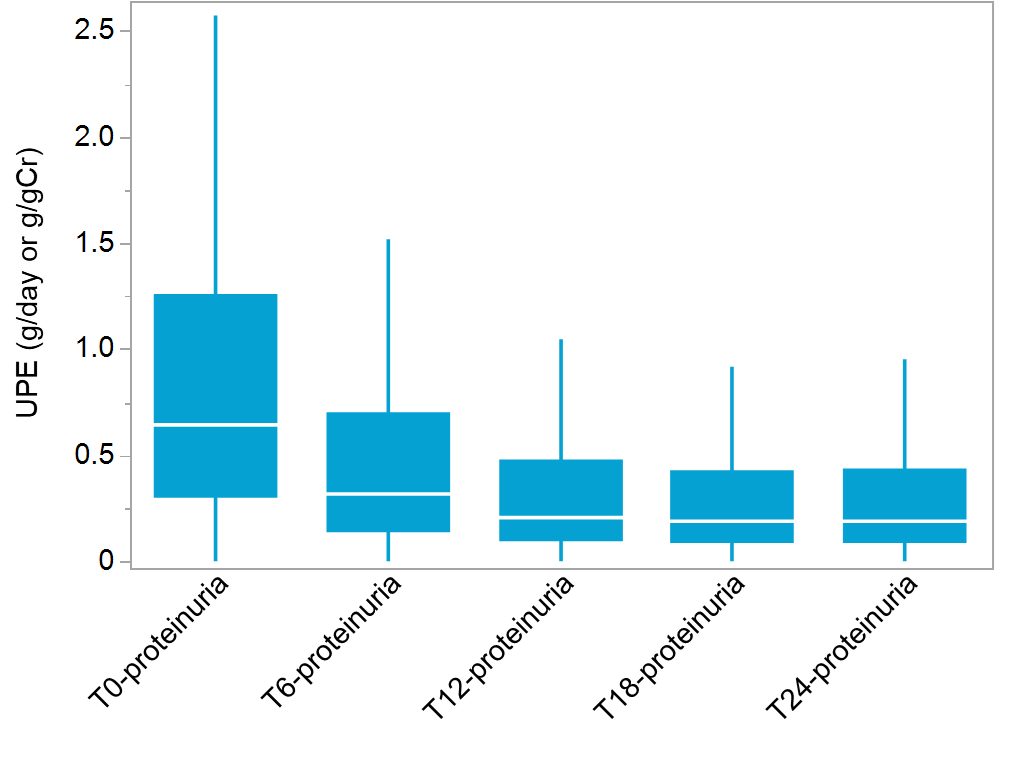
(a)


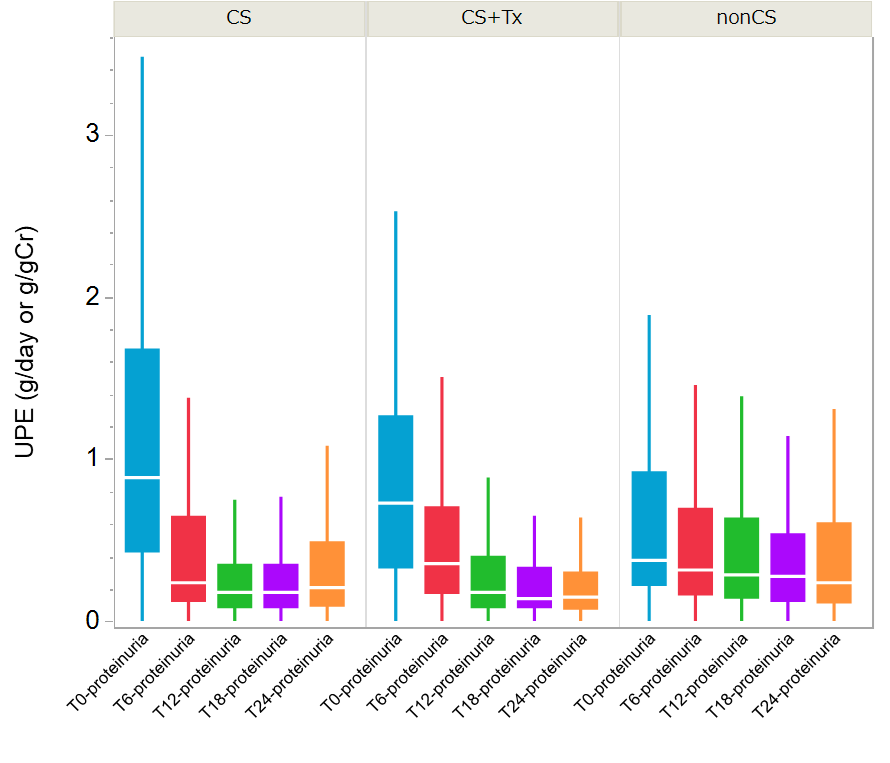
(b)

**Supplementary Fig. 2** Distribution of initiation timing for corticosteroid therapy and tonsillectomy after kidney biopsy.

Histograms illustrate the timing of corticosteroid therapy and tonsillectomy initiation. Most patients started treatment within 6 months after biopsy, whereas a few cases appear at 18 months; however, in these patients, the other treatment (corticosteroids or tonsillectomy) had already been initiated earlier.

**
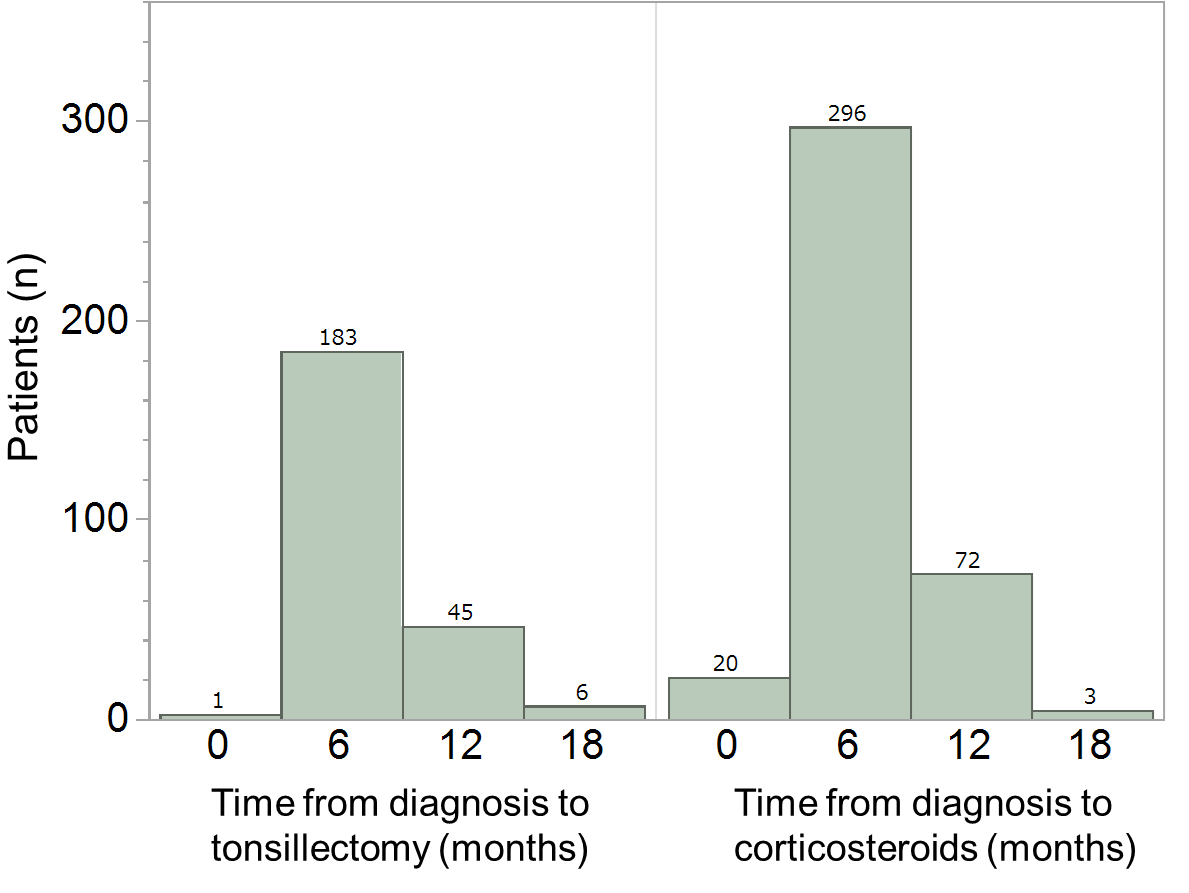
**

**Supplementary Fig. 3** Kaplan–Meier survival curves stratified by initial treatment groups and T18-proteinuria.

Kaplan–Meier curves illustrating the cumulative incidence of the primary outcome stratified by initial treatment group: (a) non-corticosteroid (non-CS), (b) corticosteroid (CS), and (c) corticosteroid plus tonsillectomy (CS+Tx). Within each group, patients were further stratified by T18-proteinuria levels (< 0.44 vs. ≥ 0.44 g/day or g/gCr). In all treatment groups, higher T18-proteinuria was consistently associated with an increased incidence of the primary outcome.


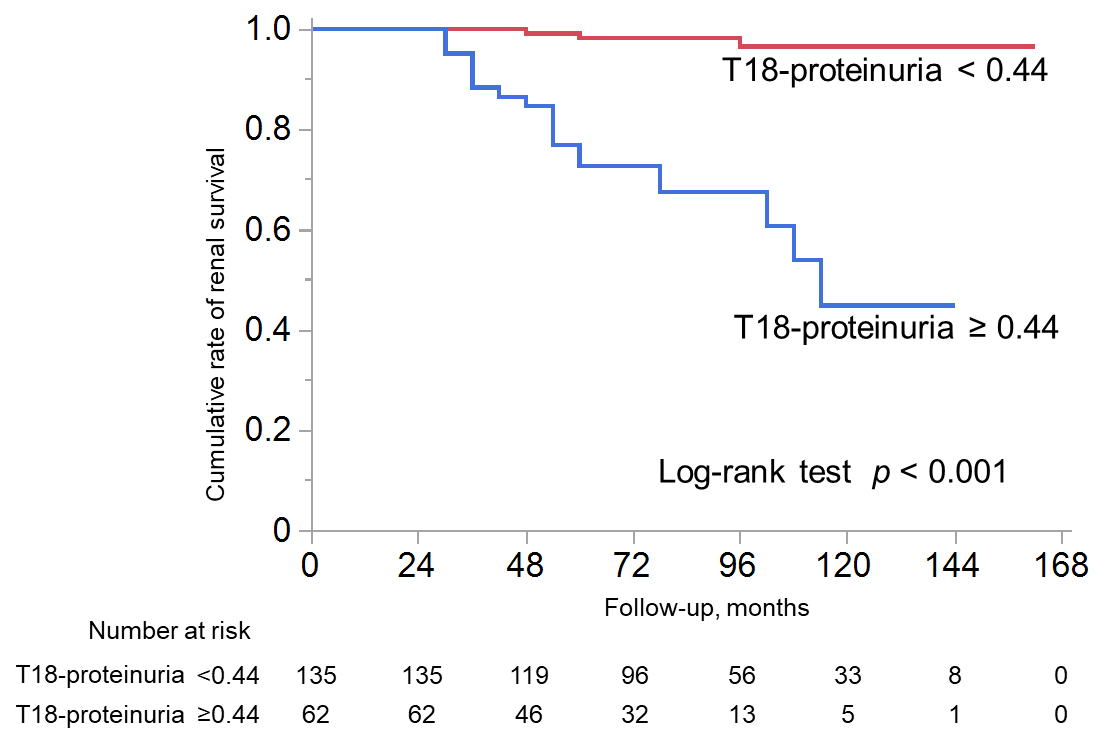
(a)


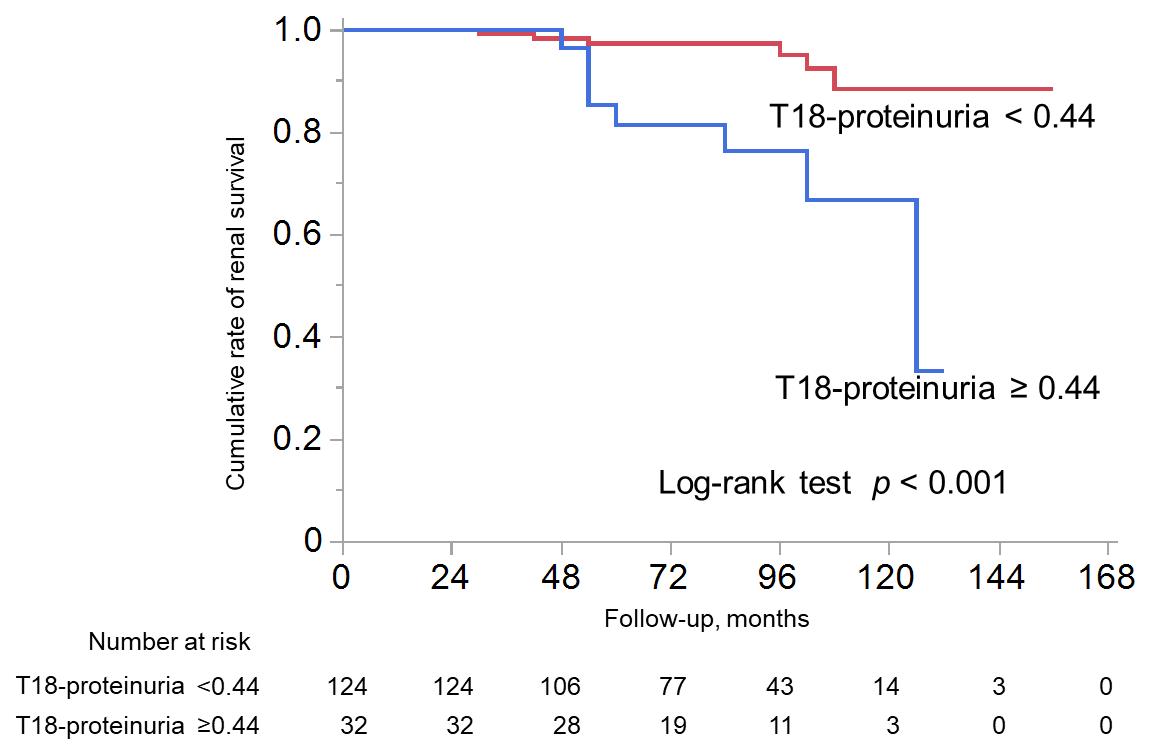
(b)


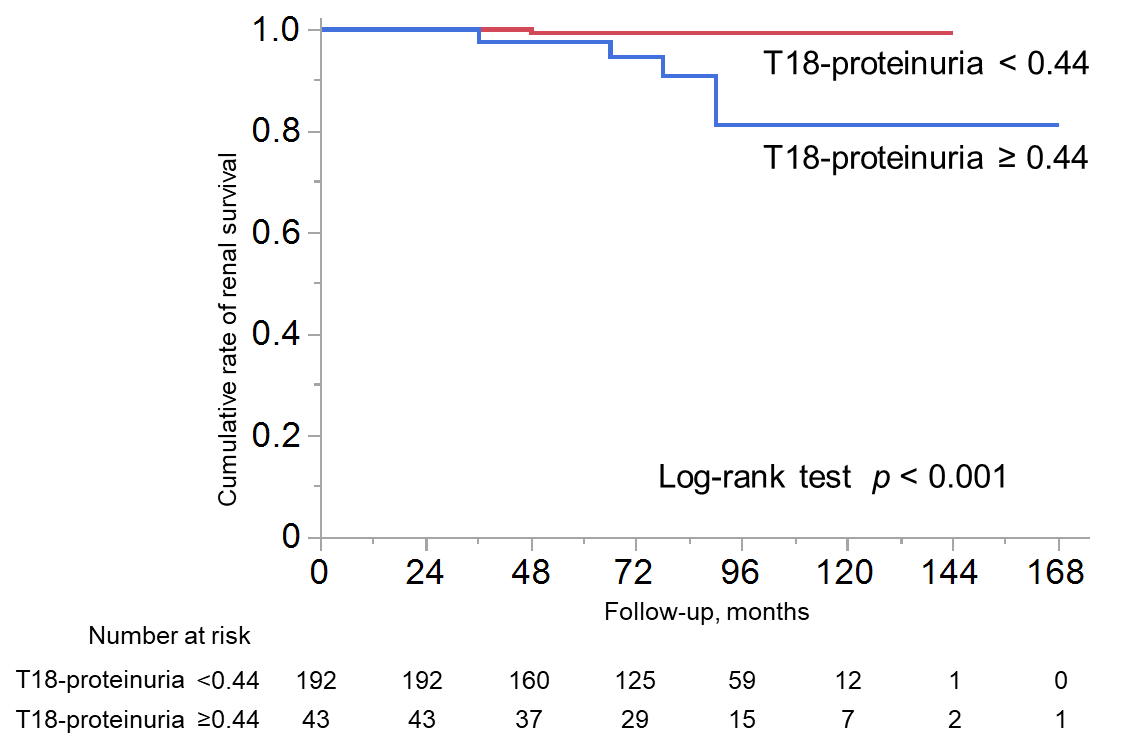
(c)

**Supplementary Fig 4** Kaplan–Meier curves stratified by T18-proteinuria for a ≥ 40% decline in eGFR.

Kaplan–Meier curves showing the cumulative incidence of a 40% or greater decline in eGFR among patients with T18-proteinuria ≥ 0.44 g/day (or g/gCr) versus those with < 0.44 g/day (or g/gCr). The high-proteinuria group showed a significantly higher incidence of the outcome (log-rank p < 0.001).

**
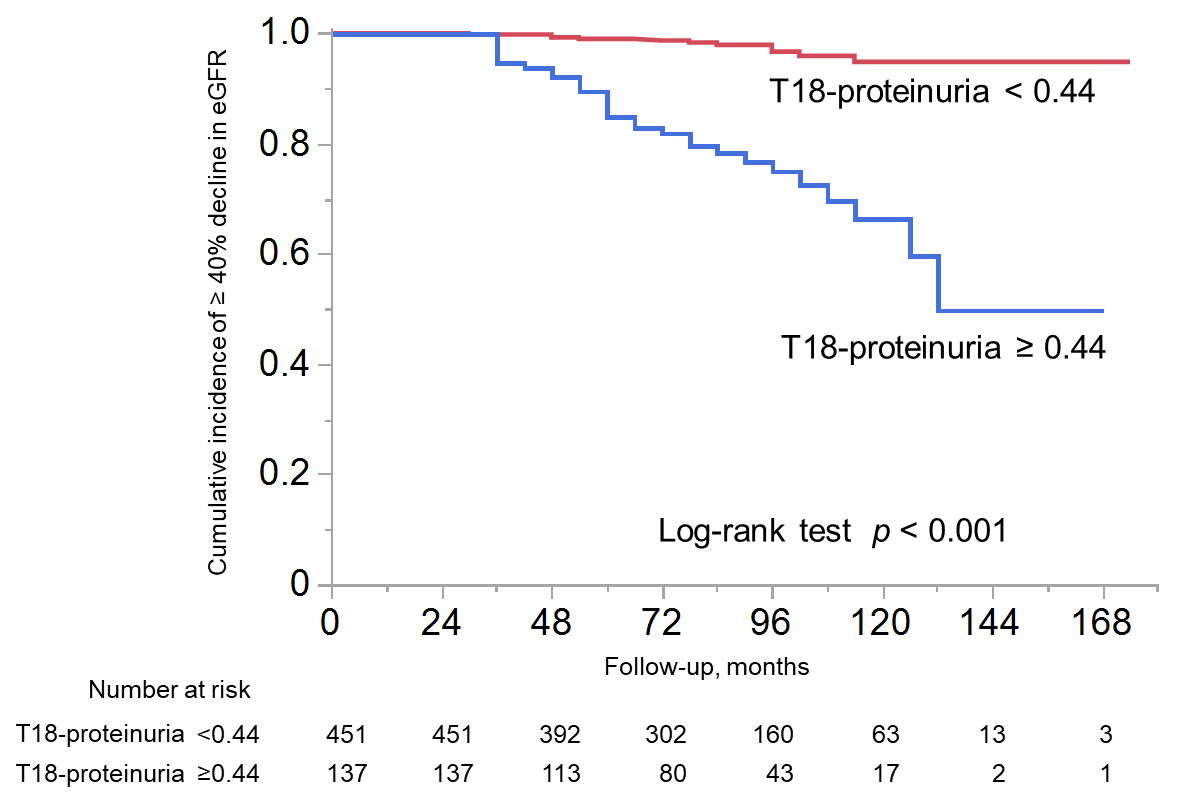
**
